# Ependymoma in a 14-week fetus with occipital encephalocele: a case report

**DOI:** 10.1515/crpm-2026-0014

**Published:** 2026-10-16

**Authors:** Mamatha K, Toshi Agarwal, Shailaja R. Bidri, Angela Susan A J, Sayandeep K. Das

**Affiliations:** Department of Pathology, BLDE (Deemed to be University), Shri B. M. Patil Medical College Hospital and Research Centre, Vijayapura, India; Department of Pathology, FH Medical College, Agra, India; Department of Obstetrics and Gynecology, BLDE (Deemed to be University), Shri B. M. Patil Medical College Hospital and Research Centre, Vijayapura, India

**Keywords:** case report, ependymoma, fetal brain tumor, histopathology, occipital encephalocele, prenatal ultrasonography

## Abstract

**Objectives:**

Ependymomas are rare glial tumors arising from ependymal cells. Most fetal ependymomas reported in the literature are identified in the third trimester. This report describes prenatal detection of an occipital encephalocele at 14 weeks’ gestation with subsequent post-termination histopathological identification of ependymoma.

**Case presentation:**

A woman in her 20s (gravida 2, para 0) underwent routine ultrasonography at 14 weeks’ gestation at a tertiary care hospital in India. Imaging showed a large occipital encephalocele with herniated disorganized brain parenchyma and associated fetal anomalies; no antenatal tumor diagnosis was made. After counseling regarding the severe cranial malformation, guarded prognosis, and management options, the parents elected medical termination of pregnancy. The fetus weighed 40 g. Histopathological examination showed monomorphic cells forming perivascular pseudorosettes and ependymal canal-like structures. Glial fibrillary acidic protein highlighted the perivascular processes; epithelial membrane antigen and S-100 were negative. The histomorphological features supported a histology-based diagnosis of ependymoma, CNS WHO grade 2. Molecular studies were not performed; therefore, molecular subgrouping was unavailable.

**Conclusions:**

At 14 weeks’ gestation, ultrasonography identified an occipital encephalocele, whereas ependymoma was identified only by post-termination histopathological examination. This case emphasizes early anatomical assessment, balanced parental counseling, and clinicopathological correlation while acknowledging uncertainty regarding the relationship between the encephalocele and the neoplasm.

## Introduction

Ependymomas are rare glial tumors arising from ependymal cells. The 2021 World Health Organization classification integrates anatomical site, histopathological features, and molecular alterations when available [1], 2].

Fetal brain tumors are rare and are usually identified later in gestation, often after macrocephaly, hydrocephalus, or an intracranial mass becomes apparent [3], 4]. Prenatal ultrasonography and fetal magnetic resonance imaging can define the anatomy and extent of fetal central nervous system abnormalities, although precise tumor classification generally requires tissue examination [4], [5], [6].

In this case, ultrasonography at 14 weeks identified an occipital encephalocele with herniated abnormal neural tissue; it did not establish a tumor diagnosis. Ependymoma was identified only on post-termination histopathological examination. The case therefore illustrates early structural detection followed by clinicopathological clarification.

## Case presentation

A woman in her 20s (gravida 2, para 0) presented for routine antenatal care at approximately 14 weeks’ gestation.

During routine antenatal care, the patient underwent ultrasonography to evaluate fetal development, which revealed a large defect in the occipital region of the fetal skull with extrusion of brain parenchyma into the amniotic cavity, consistent with an occipital encephalocele. The herniated neural tissue appeared disorganized and uncovered by bone. Associated abnormalities of the fetal facial bones were also noted, suggesting a complex craniofacial malformation. These findings raised significant concerns regarding fetal viability and the presence of a severe central nervous system anomaly. No discrete intracranial tumor was diagnosed antenatally.

Given these findings, the parents were counseled regarding the severity of the cranial malformation, the guarded fetal prognosis, the possibility of profound neurological impairment, and options for further evaluation and pregnancy management. They subsequently opted for medical termination of pregnancy. Fetal magnetic resonance imaging and prenatal genetic evaluation were not performed before termination. With parental consent, an autopsy and histopathological examination of the terminated fetus were performed.

The fetus, weighing 40 g, was received in 10 % formalin with the attached umbilical cord measuring 14 cm in length. External examination revealed protrusion of brain parenchyma from the skull, prominent globes, blackish discoloration of the left arm, skin congestion, and obliteration of the nasal cavity (Figure 1).

**Figure 1:**
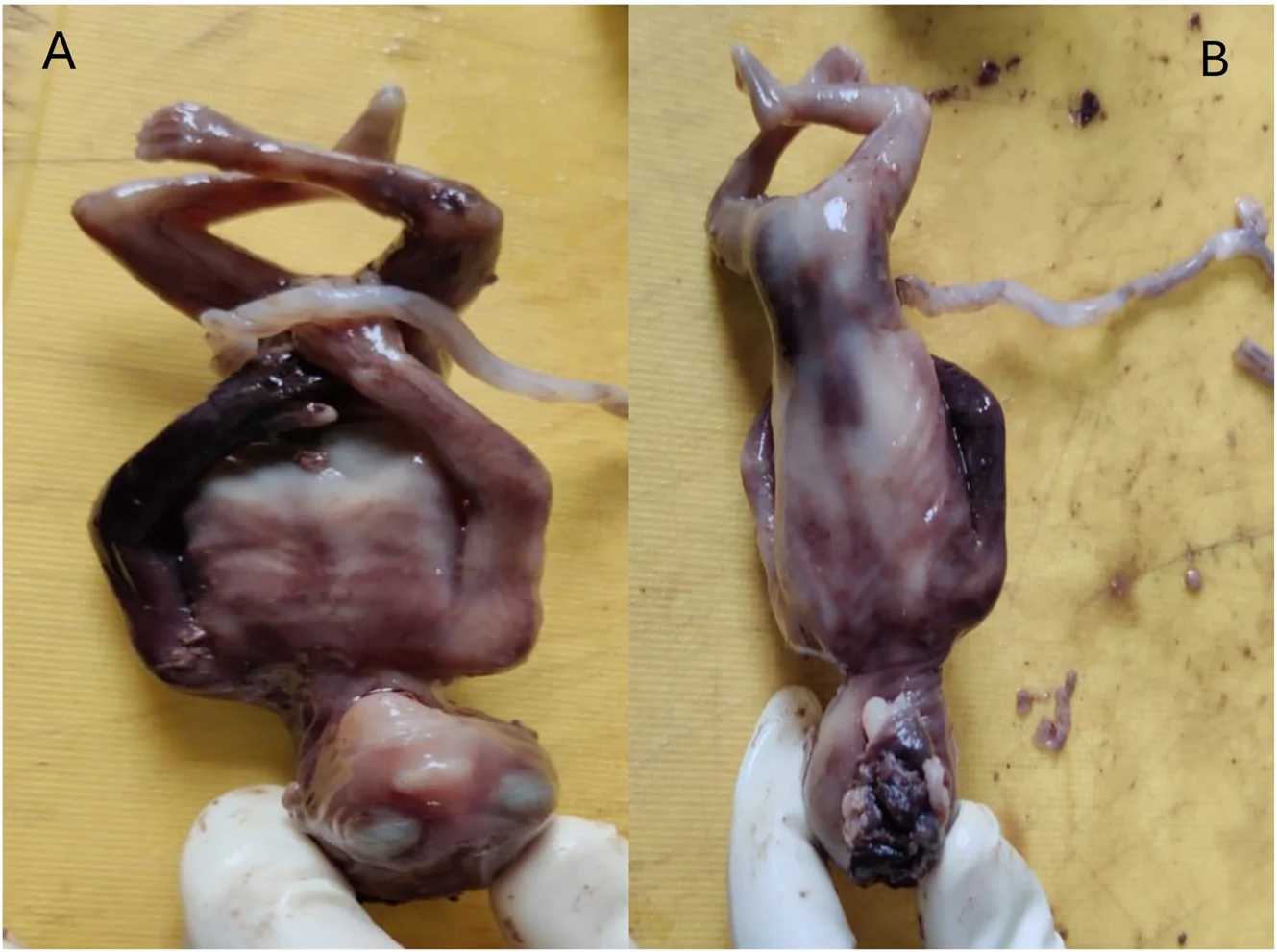
Gross photograph showing (A) prominent globes and blackish discoloration of the left arm and (B) protrusion of brain parenchyma through the occipital defect.

Brain sections were stained with hematoxylin and eosin. Microscopic examination revealed a cellular glial lesion composed of monomorphic round-to-oval cells with stippled chromatin and scant cytoplasm. The cells formed numerous perivascular pseudorosettes with intervening anuclear fibrillary zones and occasional ependymal canal-like structures (Figure 2). These architectural and cytological findings were most consistent with ependymoma.

**Figure 2:**
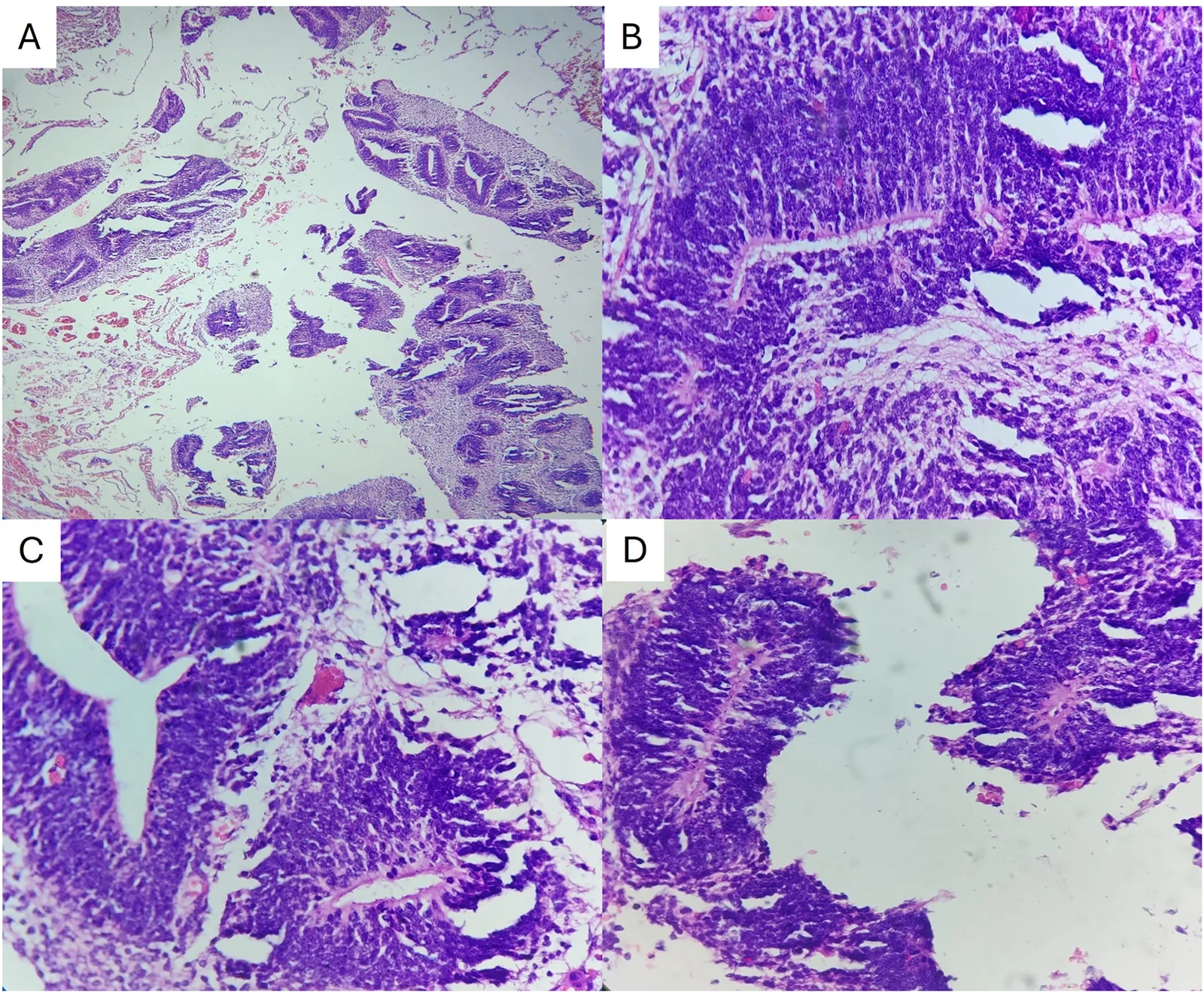
Hematoxylin and eosin staining showing (A) tumor composed of perivascular pseudorosettes and occasional ependymal canal-like structures (×40); (B) monomorphic round-to-oval tumor cells (×400); (C) stippled chromatin and scant cytoplasm (×400); and (D) perivascular anuclear zones containing delicate fibrillary processes (×400).

Immunohistochemistry showed glial fibrillary acidic protein positivity in the perivascular tumor-cell processes (Figure 3), supporting glial differentiation. Epithelial membrane antigen and S-100 were negative. The immunohistochemistry was interpreted together with the characteristic morphology and was supportive, rather than independently diagnostic, of ependymoma.

**Figure 3:**
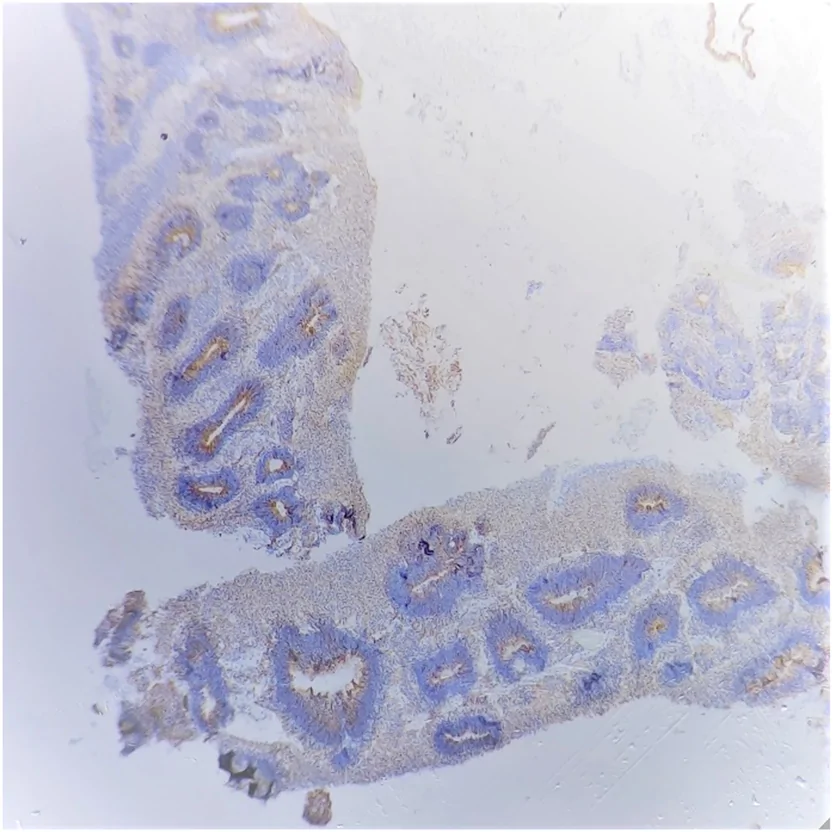
Glial fibrillary acidic protein immunohistochemistry showing positivity in the perivascular tumor-cell processes.

Based on the characteristic morphology and supportive immunophenotype, a histology-based diagnosis of ependymoma, CNS WHO grade 2, was rendered. The grade 2 designation was based on monomorphic cytology and lack of overt anaplastic morphology in the examined sections. Molecular studies were not performed; therefore, molecular subgrouping was unavailable.

The main differential diagnoses included dysplastic or reactive ependymal tissue associated with the encephalocele, an astrocytic neoplasm, an embryonal tumor, and a choroid plexus tumor. These alternatives were considered less likely because of the repetitive perivascular pseudorosettes, ependymal canal-like structures, monomorphic cytology, and glial fibrillary acidic protein-positive perivascular processes. Nevertheless, because the lesion was present in markedly dysplastic neural tissue, a developmental or reactive ependymal proliferation cannot be excluded with absolute certainty.

Written informed consent for termination, autopsy, and publication of clinical details and images was obtained from the patient before the procedure. As this is a single-patient case report, no statistical analyses were performed. The manuscript was prepared in accordance with the CARE case report guidelines [7].

## Discussion

Fetal brain tumors are rare and are usually recognized later in pregnancy. In the review by Isaacs, five ependymal tumors were identified among 154 fetal brain tumors, and most fetal brain tumors were detected in the third trimester [3]. The present case is unusual because a severe occipital cranial anomaly was detected at 14 weeks; however, the antenatal finding was an occipital encephalocele, not a classified tumor.

The ependymoma diagnosis was established only after histopathological examination. Recognized germline predisposition accounts for a minority of pediatric ependymomas [8], and no molecular or germline testing was performed in this case. Therefore, this single observation does not allow estimation of recurrence risk for future pregnancies. Genetic counseling and psychosocial support may nevertheless be offered after a pregnancy affected by a severe fetal anomaly.

Detailed neurosonography and, when feasible at an appropriate gestational age, fetal magnetic resonance imaging may help define the anatomy of an encephalocele and associated abnormalities [4], [5], [6]. Genetic evaluation may be considered individually when multiple structural anomalies are present. The available evidence does not support routine NF2 testing solely on the basis of this case.

At early gestational ages, fetal central nervous system anatomy and markedly dysplastic herniated neural tissue can be difficult to characterize. Prenatal imaging is important for defining structural abnormalities, whereas histopathological examination remains essential for tumor classification and diagnostic clarification.

The morphology in this case – perivascular pseudorosettes, ependymal canal-like structures, and glial fibrillary acidic protein-positive perivascular processes – supported ependymoma. Epithelial membrane antigen and S-100 negativity were not considered independently confirmatory. Under the current World Health Organization framework, ependymomas are classified using anatomical site, histopathological features, and molecular findings when available [1], 2]. Because molecular studies were not performed, the diagnosis remains histology based and a molecular subgroup could not be assigned.

The relationship between the encephalocele and the ependymoma cannot be determined from this single case. The neoplastic process may have contributed to the malformation, may have arisen within dysplastic herniated neural tissue, or may have been an incidental microscopic finding. The lesion should therefore not be presented as unequivocally causal for the encephalocele.

Limitations include the absence of fetal magnetic resonance imaging, prenatal genetic evaluation, and molecular tumor studies; the limited immunohistochemical panel; and the inability to establish whether the ependymoma caused, arose within, or was incidental to the encephalocele. Nevertheless, the characteristic morphology and supportive immunophenotype permitted a cautious histology-based diagnosis.

## Conclusions

At 14 weeks’ gestation, ultrasonography identified a severe occipital encephalocele; ependymoma was identified only after post-termination histopathological examination. This case emphasizes careful early fetal anatomical assessment, balanced parental counseling, and clinicopathological correlation. The causal relationship between the encephalocele and the neoplasm remains uncertain.
